# Study on the Ventilation Method to Maintain the PM_10_ Concentration in a Subway Cabin below 35 μg/m^3^

**DOI:** 10.3390/toxics10100560

**Published:** 2022-09-25

**Authors:** Eun-Seo Choi, Se-Jin Yook, Minjeong Kim, Duckshin Park

**Affiliations:** 1 School of Mechanical Engineering, Hanyang University, Seoul 04763, Korea; 2 Artificial Intelligence Railroad Research Department, Korea Railroad Research Institute, Uiwang-si 16105, Gyeonggi-do, Korea; 3 Transportation Environmental Research Department, Korea Railroad Research Institute, Uiwang-si 16105, Gyeonggi-do, Korea

**Keywords:** subway cabin, PM_10_, HVAC, filter efficiency, indoor air quality

## Abstract

The city of Seoul will limit the maximum particulate matter (PM_10_) concentration to ≤35 μg/m^3^ (from 2024). Herein, a numerical parametric study was conducted on the PM removal efficiency of the heating, ventilation, and air conditioning (HVAC) filters installed in the ceiling of subway cabins. The PM_10_ concentration distribution was explored according to the flow rate and flow rate ratio of the air introduced into the cabin. Under the current ventilation conditions of the subway train HVAC system, the PM_10_ concentration was highest in the cabin central area where exhaust outlets are located and decreased toward both ends of the cabin. The indoor airflow was improved and the PM_10_ concentration was reduced by increasing the flow rate of the supplied air at both ends of the cabin while decreasing it in the central area. It was found that the strengthened PM_10_ concentration criterion of Seoul can be met by increasing the ventilation flow rate to 700 CMH (currently, 500 CMH) and the filter efficiency to 85% (currently, 70%) while maintaining the current flow rate ratio. These results are expected to be used as important reference data for reducing the PM concentration in subway cabins and thereby improving indoor air quality.

## 1. Introduction

The causes of presence of particulate matter (PM) inside subway cabins include outdoor and indoor factors. PM is discharged to the atmosphere during the combustion of fossil fuels. An example of the outdoor factor is the introduction of PM from the atmosphere into underground tunnels [1]. Meanwhile, in underground tunnels, PM is generated by the frictional wear between the rails and wheels and between pantographs and electric wires. An example of the indoor factor is the continuous scattering of a large amount of PM generated inside tunnels due to train wind caused by traveling subway trains [2]. Platform screen doors prevent the air inside the tunnel from being spread to the platform, but they can increase the PM concentration inside the tunnel by accumulating the generated pollutants there [3]. An increase in the PM concentration inside subway tunnels has an impact on the indoor air quality of subway cabins, thereby affecting the health of passengers who spend approximately 75% of their commute time in cabins [4]. In particular, respirable PM has a harmful effect on health because it can penetrate deeply into the lungs. As a result, PM may increase mortality by causing the degradation of the lung function, cardiovascular diseases, and respiratory diseases [5,6]. Therefore, the city of Seoul decided to limit the maximum PM concentration in subway cabins to maintain it below 35 μg/m^3^ from 2024. Accordingly, various studies have been conducted to improve indoor air quality inside subway cabins.

To reduce the PM concentration in the subway environment, various filters have been used in ventilation facilities, such as in cabins, tunnels, and underground stations and on platforms, and their effects have been investigated. In a previous study, a subway cabin air purifier (SCAP) was installed in the ceiling of a cabin, and the PM concentration was measured. Therein, high blocking efficiency against PM_10_ and PM_2.5_ particles was observed when the SCAP was used [7]. Further, the PM-blocking efficiency was examined according to the height and angle of the blocking device after its installation in the heating, ventilation, and air conditioning (HVAC) system to prevent the PM introduction from underground tunnels to subway cabins. The highest blocking efficiency was observed at a height of 80 mm and an angle of 90° [8]. In another study, magnet–magnet (MM) and magnet–cascade (MC) filters were installed on a subway platform, and the PM_10_ concentration was measured. The removal efficiency of the MM and MC filters was 27% and 40.5%, respectively [9]. Next, the effects of using electret filters, electret bundle filters, and electret pleated filters in combination with the existing filters used in tunnel ventilation facilities were explored. The PM_10_ and PM_2.5_ removal efficiencies were 85% and 55%, respectively, when prefilters were used in combination with electret pleated filters [10].

Various studies have been conducted to improve indoor air quality in the subway environment in ways other than using filters. For example, an exhaust system was installed in the space under the stairs that led to the platform and waiting room. Consequently, the ventilation efficiency was improved by 16.5% [11]. Many studies were conducted to reduce toxic materials, such as CO_2_, TVOCs, and VOCs, in addition to studies on the reduction of the PM concentration in subway cabins, tunnels, and underground stations and on platforms. In one study, the operation of mechanical exhaust fans installed on both sides of a subway train was monitored, and the CO_2_ concentration generated in the cabin during subway operation was observed. The CO_2_ concentration in the cabin increased to 5000 ppm if the number of passengers exceeded the capacity, reaching up to 200% of the cabin design limit, but it decreased to 1500 ppm when the mechanical exhaust fans were operated [12]. Furthermore, changes in the concentration of TVOCs were examined while the flow velocity of the air blower in the subway train was changed in the 0.2–0.7 m/s range. It was found that the TVOC concentration in the respiratory area of passengers decreased as the flow velocity increased [13].

As mentioned above, many studies have been conducted to improve the collection efficiency of dust collectors, such as filters, or ventilation airflow in a space in order to reduce the concentrations of various toxic materials, including PM, in the subway environment. However, few studies have been conducted on the filter efficiency or ventilation methods in cabins required to meet the PM_10_ concentration maximum (≤35 μg/m^3^) recently reduced for subway cabins. As such, in this study, the indoor PM_10_ concentration in the subway trains operated on Seoul Metro Line 5 according to the efficiency of the filter applied to the HVAC system equipped with a SCAP was identified through simulation. As a result, the filter efficiency satisfying the reduced indoor PM_10_ concentration maximum of Seoul was determined. Furthermore, efforts were made to determine an optimal flow rate ratio so as to decrease the average indoor PM_10_ concentration by changing the flow rate ratio through the 16 supply inlets installed in the cabin ceiling. Meanwhile, the total ventilation flow rate of the HVAC system equipped with a SCAP was fixed. In addition, a method to decrease the required filter efficiency by increasing the existing flow rate of the HVAC system equipped with a SCAP was proposed.

## 2. Materials and Methods

**Figure 1:**
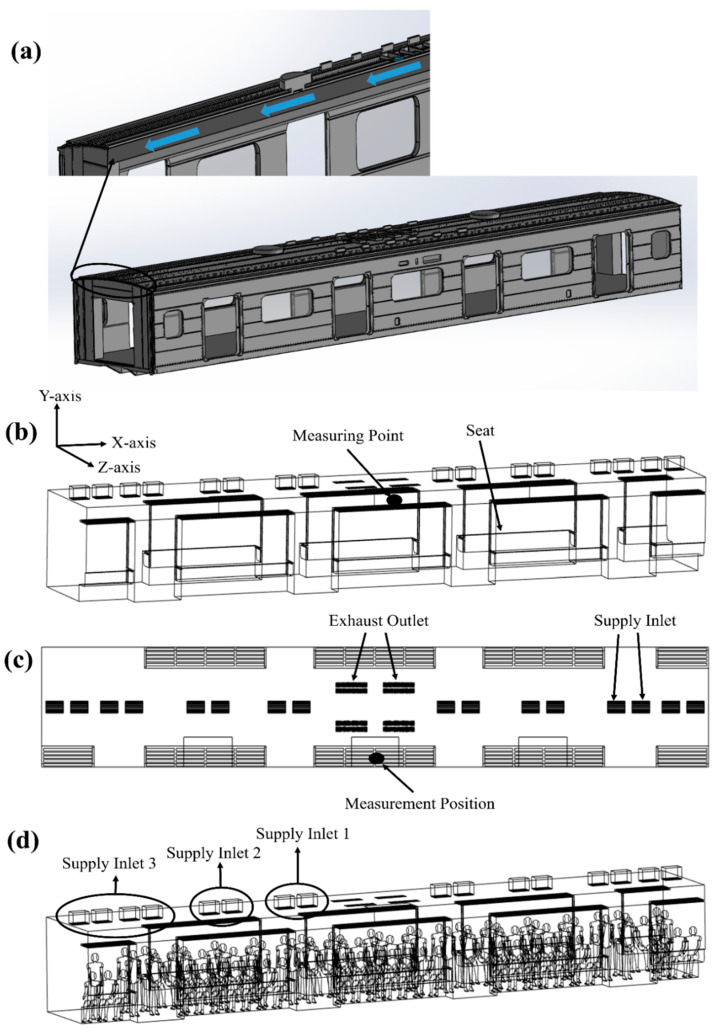
Schematic of a subway cabin. (**a**) HVAC air supply duct; (**b**) simplified geometry of the supply inlets and exhaust outlets; (**c**) positions of the supply inlets and exhaust outlets (top view); and (**d**) supply inlet groups.

Figure 1 shows a schematic of a subway cabin operated on Seoul Metro Line 5. It has a length of 18.92 m, width of 3.2 m, and height of 2.74 m. In the ceiling of the cabin, two HVAC systems equipped with a SCAP are installed symmetrically in the longitudinal direction with respect to the center of the cabin, and the flow rate of each HVAC system is 250 CMH [14]. Four return air outlets suck the indoor air in the center, and 16 supply air inlets are arranged in the longitudinal direction of the cabin. In other words, two return air outlets and eight supply air inlets are connected to each HVAC system. The circulation method of this HVAC system equipped with a SCAP is as follows. A total of 30% of the airflow sucked from the cabin is discharged to the tunnel, and the remaining 70% are recirculated. The 70% recirculated air is combined with the 30% flow newly introduced from the tunnel and is introduced into the cabin through filters with 70% efficiency [15].

Figure 1a shows the duct that conveys the supply air from the HVAC system equipped with a SCAP into the cabin. Because analysis that includes a duct with a complex geometry significantly increases the number of grids and the computation time, only the flow of the duct was separately analyzed. As shown in Figure 1b, the supply inlets through which the air from the duct flows into the cabin were simplified. Further, the flow rate of each supply inlet obtained from the duct flow analysis results was applied to the simplified geometry [16,17]. Figure 1c shows the four exhaust outlets in the center and the 16 supply inlets in the longitudinal direction. As shown in Figure 1d, the eight supply inlets connected to each HVAC system equipped with a SCAP were divided into three areas (supply inlets 1, 2, and 3). Table 1 shows the measurement results obtained by Jeong et al. [18]. Firstly, it lists the average, maximum, and minimum values of the PM_10_ concentration in a subway cabin of Seoul Metro Line 5 measured at the position on the shelf in the center of the cabin (measurement position in Figure 1). Secondly, it lists the PM_10_ concentration in a tunnel during non-rush hours. Considering non-rush hours, the number of passengers in the cabin was assumed to be 51 when there were only seated people and 88 when there were seated and standing people. As the number of grids increased rapidly with the number of people, up to 88 people could be considered in this study. ANSYS FLUENT Release 19.0, a commercial CFD code, was used to predict the airflow and PM_10_ concentration distribution in the subway cabin. The flow of air was assumed to be three-dimensional, steady, incompressible, and turbulent. The buoyancy effect due to heat generation by the passengers was assumed to be negligible [19]. The continuity equation and the momentum equations were solved for the flow analysis, and the *k*–*ε* turbulence model was used for turbulence analysis [16,17,20]. QUICK was set as the momentum equation scheme, SIMPLEC—as the pressure-velocity coupling scheme, and PRESTO—as the pressure interpolation scheme. As for the boundary conditions for the flow analysis, the no-slip condition was set for all the surfaces of the cabin and people, velocity inlet condition for the supply air inlets, and pressure outlet condition for the return air outlets. The total flow rate of the air sucked through the exhaust outlets in the center of the cabin was 500 CMH. A total of 30% of it was discharged to the tunnel, and the remaining 70% were recirculated. In order to predict the PM_10_ concentration, the user-defined scalar transport equation provided in the FLUENT was solved as follows [21]:

(1) $$\frac{\partial \rho \phi }{\partial t}+\frac{\partial }{\partial x_{i}}\left(\rho u_{i}\phi -\Gamma \frac{\partial \phi }{\partial x_{i}}\right)=S_{\phi }$$

Here, *ρ* is the density of air, ϕ is the concentration of PM_10_, *t* is the time, $x_{i}$ is the coordinate, $u_{i}$ is the velocity of air, Γ is the diffusion coefficient, and $S_{\phi }$ is the source term. The 70% recirculated air was combined with the 30% air newly introduced from the tunnel, and then it was introduced into the cabin through filters with 70% efficiency and the supply inlets [14,15]. A user-defined function (UDF) was used to reflect the efficiency of the filter when such a ventilation flow was implemented in the simulation.

**Figure 2:**
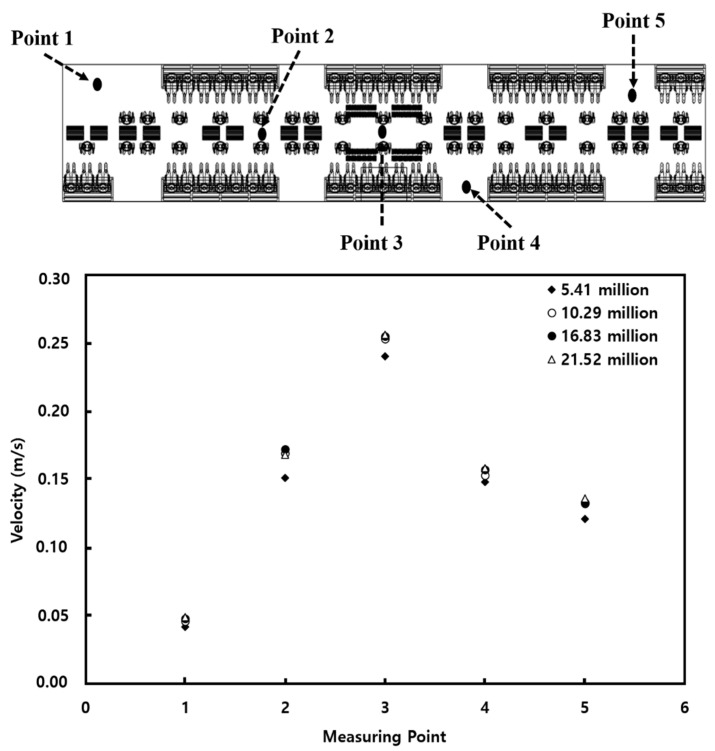
Grid independence test result.

Based on the method employed by Cao et al. [22] and Li et al. [23], a grid independence test was conducted while changing the number of tetrahedral grids from approximately 5.41 to 21.52 million. As illustrated in Figure 2, airflow velocities were monitored at five different points in the cabin and compared with those obtained from the mesh system having the largest number of grids (21.52 million). When the number of grids was varied as 5.41, 10.29, and 16.83 million, the maximum relative error was 6.8%, 1.8%, and 1.6%, respectively. Therefore, by considering the computation time and accuracy, the number of grids was determined as 10.29 million.

Lin et al. [24] evaluated air quality in offices according to the positions of supply inlets and exhaust outlets and confirmed that indoor air quality was improved by changing their positions. It was impossible to change the positions of the supply inlets and exhaust outlets installed in the subway cabin. Therefore, by referring to the results obtained by Lin et al. [24], changes in the PM_10_ concentration in the cabin were examined by changing the ratio of the airflow rates through each supply inlet of the HVAC system equipped with a SCAP. That is, the ratio of the airflow rates through supply inlets 1, 2, and 3 was changed with respect to the flow rate of each HVAC system. Table 2 lists all cases considered in this study, with the parameters of total ventilation flow rate, filter efficiency, and flow rate ratio. The reference ratio of the air introduced through supply inlets 1, 2, and 3 was 20:20:60. In an effort to lower the PM_10_ concentration in the cabin, only for some selected cases, the filter efficiency varied as 70, 80, 85, and 90%, and the total ventilation flow rate through the two HVAC systems was increased from 500 to 700 CMH, i.e., the ventilation flow rate through each HVAC system was varied as 250, 300, and 350 CMH. For every HVAC ventilation flow rate, the flow rate ratios were applied as listed in Table 2. For example, when the flow rate ratio was 20:20:60, the flow rates of the air injected from supply inlets 1, 2, and 3 were 50, 50, and 150 CMH, respectively, for the HVAC ventilation flow rate of 250 CMH; and they were 70, 70, and 210 CMH, respectively, for the HVAC ventilation flow rate of 350 CMH.

## 3. Results and Discussion

### 3.1. Model Validation

**Figure 3:**
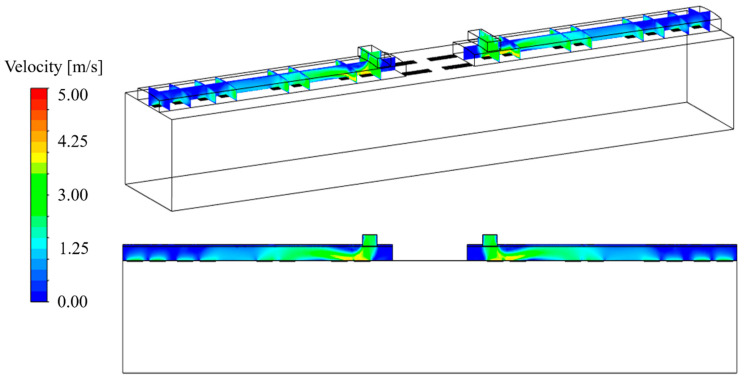
Air velocity distribution in the HVAC air supply duct.

Figure 3 shows the airflow velocity distribution in the duct of the HVAC systems. As can be observed for the flow rate of each HVAC system equipped with a SCAP (250 CMH), the recirculation of 70% of the air sucked through the two exhaust outlets in the center of the cabin ceiling was well-simulated. Furthermore, its introduction into the cabin through the eight supply inlets after being combined with the 30% air newly supplied from the tunnel was well-simulated. The ratio of the airflow rates introduced into the cabin through supply inlets 1, 2, and 3 was found to be approximately 20:20:60.

**Figure 4:**
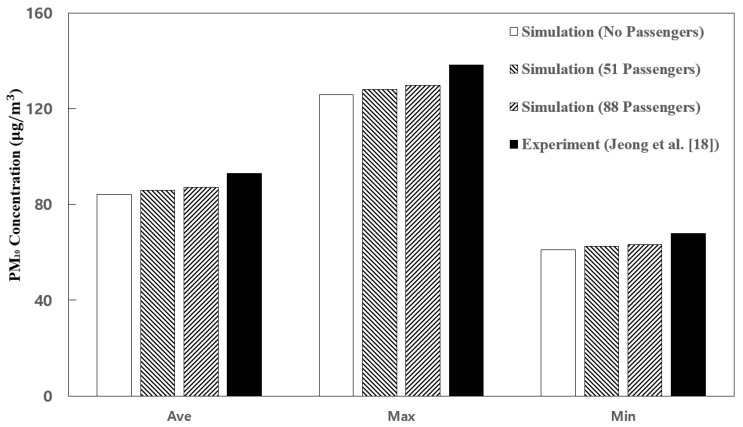
Comparison of the PM_10_ concentration at the measurement point inside the subway cabin between the simulation prediction results of this study and the measurement data obtained by Jeong et al. [18].

**Figure 5:**
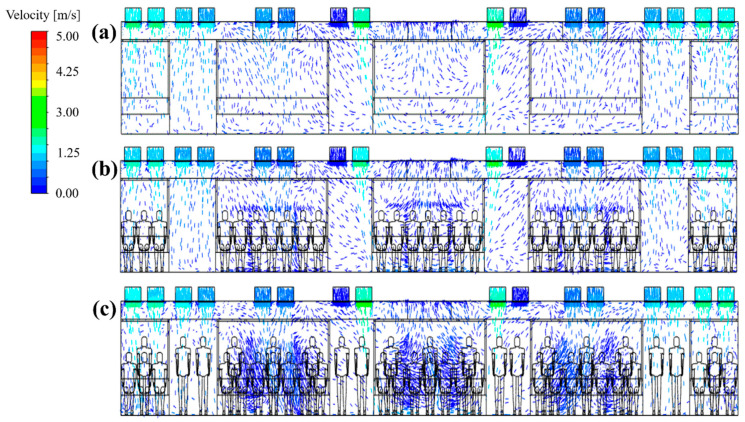
Airflow velocity vectors in the central section of the cabin according to the number of passengers (total ventilation flow rate: 500 CMH). (**a**) No people; (**b**) 51 people (all seated); and (**c**) 88 people (51 seated and 37 standing).

Figure 4 compares the simulation prediction results of this study with the experimental data of Jeong et al. [18] shown in Table 1. The average, maximum, and minimum values of the PM_10_ concentration at the position on the shelf in the center of the cabin (measurement position in Figure 1) were compared. Here, the total flow rate of the two HVAC systems equipped with a SCAP was 500 CMH, and the efficiency of the filters used in the HVAC systems was set to 70%. As Jeong et al. [18] did not specify the number of passengers in the cabin at the time of the experiment, the number of passengers in the simulation of this study was assumed to be 0, 51 (all seated), and 88 (51 seated and 37 standing). First, in the simulation results of this study, the PM_10_ concentration in the cabin showed a tendency to increase as the number of passengers was increased. To identify the cause of this result, the indoor airflow was analyzed according to the number of passengers. The airflow velocity vectors in the central section of the cabin are shown in Figure 5. When the number of passengers was zero, the airflow circulation in the cabin from the supply inlets to the exhaust outlets was found to be efficient. However, according to Posner et al. [25], indoor obstacles have a significant impact on the flow pattern and flow residence time. Therefore, as shown in Figure 5, the airflow circulation was inefficient owing to people as the number of passengers was increased. It appears that the PM_10_ concentration in the cabin increased because airflow became complicated in the center of the cabin where many people were concentrated near the center. Thus, the air was not efficiently exhausted from the supply inlets to the exhaust outlets. In this study, up to 88 people were considered as the number of passengers because of the limited number of meshes. If the number of passengers considered in the numerical analysis is further increased, the simulation results are expected to be closer to the experimental values. In addition, the simulation of this study did not consider the amount of PM generated by the respiration volume of each person and the movement of people. However, people’s breathing, conversation, coughing, and movement could have affected the PM_10_ concentration in the cabin in the experiment of Jeong et al. [18]. Nevertheless, as shown in Figure 4, the PM_10_ concentrations predicted in the simulation of this study were found to be similar to the measurement results obtained by Jeong et al. [18]. This sufficiently verified the numerical analysis model of this study. Based on these verification results, the PM_10_ concentration in the cabin was analyzed through the numerical analysis method while the filter efficiency, supply inlet flow rate ratio, and supply flow rate were changed. In addition, the number of passengers in the cabin was assumed to be 88.

### 3.2. Effect of the Filter Efficiency on the PM_10_ Concentration Distribution (Total Flow Rate: 500 CMH)

**Figure 6:**
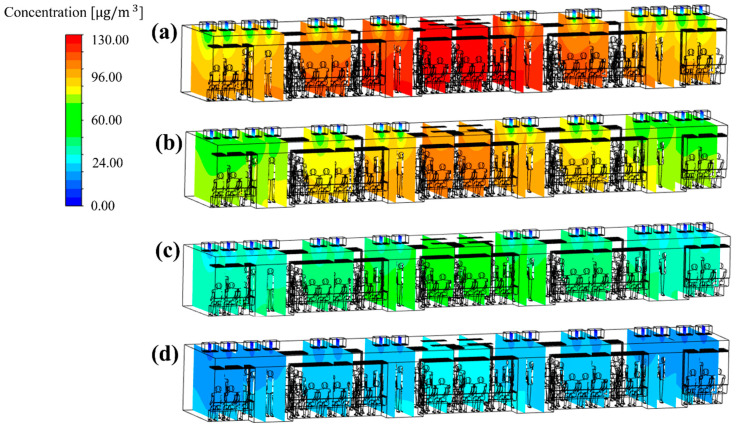
PM_10_ concentration distribution inside the subway cabin according to the efficiency of filters used in the HVAC system (total ventilation flow rate: 500 CMH): (**a**) 70% (case 1); (**b**) 80% (case 2); (**c**) 85% (case 3); and (**d**) 90% (case 4).

**Figure 7:**
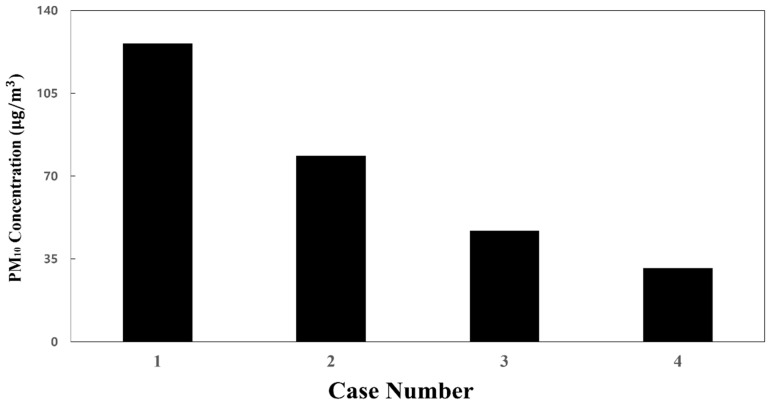
Average PM_10_ concentration in the cabin according to filter efficiency (a total ventilation flow rate of 500 CMH and flow rate ratio of 20:20:60 applied).

Figure 6 shows the results of predicting the PM_10_ concentration in the cabin through simulation when the efficiency of the filters used in the HVAC systems equipped with a SCAP was assumed to be 70% (case 1), 80% (case 2), 85% (case 3), and 90% (case 4). Here, from the duct analysis results in Figure 3, the ratio of the airflow rates through supply inlets 1, 2, and 3 was set to 20:20:60 for 250 CMH, which is the flow rate of each HVAC system equipped with a SCAP. It was found that the PM_10_ concentration in the cabin was highest in the central area where the exhaust outlets were located and gradually decreased toward both ends of the cabin. According to Zhao et al. [13], indoor air quality can be improved by increasing the flow rate of the supplied air. Similar trends were also observed in the simulation results of this study. It appears that the PM_10_ concentration was relatively low at both ends of the cabin (supply inlet 3) owing to the high flow rate of the supplied air. In contrast, the PM_10_ concentration was relatively high in the central space of the cabin (supply inlet 1) owing to the low flow rate of the supplied air and the exhaust outlets located nearby. Meanwhile, Figure 7 shows the average PM_10_ concentration in the cabin for cases 1–4. As the grade of the filter used in the HVAC systems was increased, i.e., as the removal efficiency of the filter increased, the PM_10_ concentration in the cabin was found to decrease. When the filters with 90% efficiency were applied to the current subway train operating conditions of Seoul Metro Line 5 (i.e., case 4), it was predicted that the average PM_10_ concentration in the cabin could be maintained below 35 μg/m^3^ during non-rush hours. However, the higher the filter efficiency, the greater the operating costs. Therefore, depending on the business strategy, it may be required to find other ways to lower the PM_10_ concentration in the cabin.

### 3.3. Effect of the Flow Rate Ratio on the PM_10_ Concentration Distribution (Total Flow Rate: 500 CMH)

**Figure 8:**
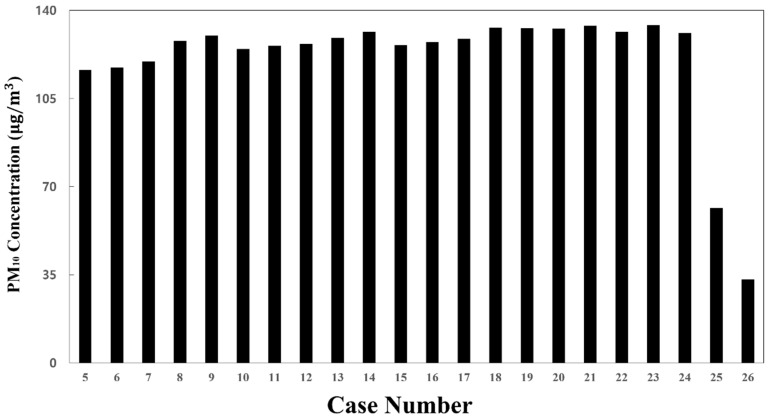
Average PM_10_ concentration in the cabin for each flow rate ratio with respect to the total ventilation flow rate of 500 CMH (filter efficiency of 70% applied for cases 5–24, 80% for case 25, and 85% for case 26).

Figure 8 compares the average PM_10_ concentration in the cabin for cases 5–24, where the flow rate ratio was varied while the total ventilation flow rate and filter efficiency were fixed to 500 CMH and 70%, respectively. In this instance, the number of people in the cabin was assumed to be 88. Overall, the PM_10_ concentration in the cabin decreased as the flow rate of supply inlet 3 was increased, and it increased as the flow rate of supply inlet 1 was increased.

**Figure 9:**
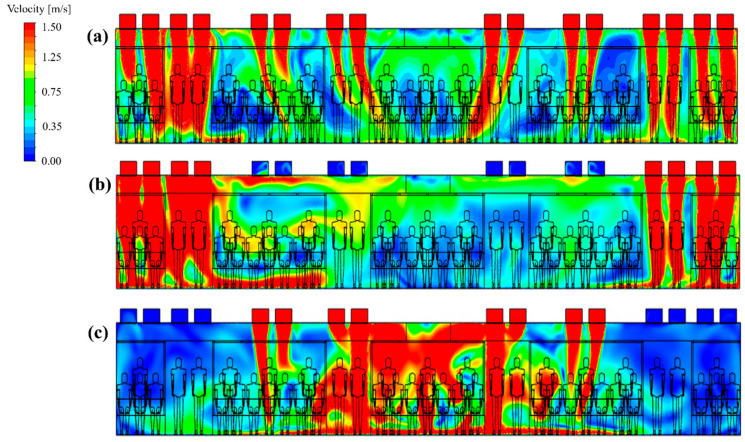
Airflow velocity distribution in the central section of the cabin according to the selected flow rate ratios (a total ventilation flow rate of 500 CMH and filter efficiency of 70% applied). (**a**) Case 1; (**b**) case 5; and (**c**) case 23.

**Figure 10:**
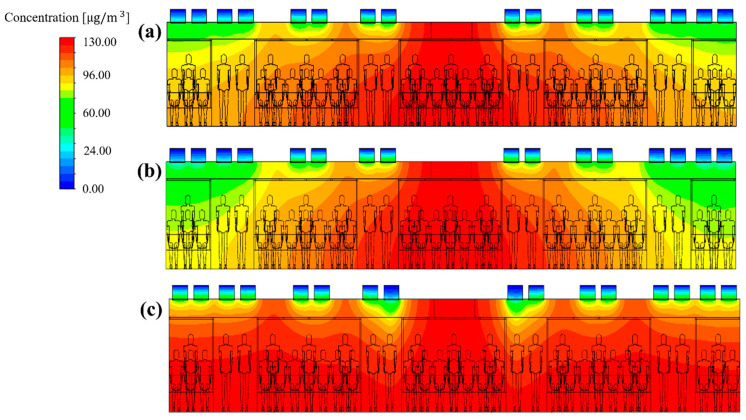
PM_10_ concentration distribution in the central section of the cabin according to the selected flow rate ratios (a total ventilation flow rate of 500 CMH and filter efficiency of 70% applied). (**a**) Case 1; (**b**) case 5 and (**c**) case 23.

To identify the cause of the difference in the PM_10_ concentration distribution in the cabin depending on the flow rate ratio, the flow velocity distribution (Figure 9) and the concentration distribution (Figure 10) were analyzed for representative cases. Figure 9a–c shows the airflow velocity distribution in the central section of the cabin for case 1 (reference model), case 5 (in which the lowest PM_10_ concentration was predicted among cases 5–24), and case 23 (in which the highest PM_10_ concentration was predicted among cases 5–24), respectively. It should be noted that the filter efficiency was 70% for cases 1, 5, and 23. Figure 10a–c shows the concentration distribution in the same section for the same cases. First, the velocity and concentration distribution are not symmetric on both sides in each figure. This is because in the *z* direction the seats were located on both sides at the right end of the cabin, but only on one side at the left end (Figure 1b,c, respectively). Figure 9b shows that the flow rate of supply inlet 3 was increased compared with that of the reference model (Figure 9a). It can be seen that the air flows more efficiently from the supply inlets to the exhaust outlets. This decreases the PM_10_ concentration at both ends of the cabin compared to that of the reference model (Figure 10a), as shown in Figure 10b. However, when the flow rate of supply inlet 1 was increased (Figure 9c), the air could not efficiently flow at both ends of the cabin, and complex turbulence was formed in the central area of the cabin. This increased the PM_10_ concentration throughout the cabin as the clean air introduced into the cabin through the supply inlets was mixed with the airflow around the exhaust outlets, as shown in Figure 10c. Based on these results, it was deemed desirable to increase the flow rate of supply inlet 3 located at both ends of the cabin and decrease the flow rate of supply inlet 1, which is close to the exhaust outlets. In this way, the PM_10_ concentration can be reduced because of efficient airflow throughout the cabin.

**Figure 11:**
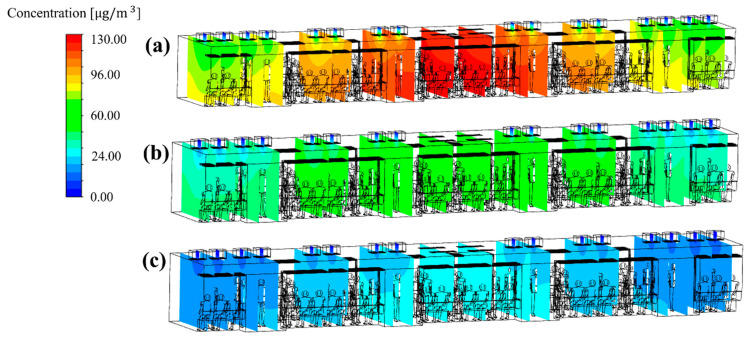
PM_10_ concentration distribution in the subway cabin according to the filter efficiency for the flow rate ratio of 0:0:100 (total ventilation flow rate: 500 CMH): (**a**) 70% (case 5); (**b**) 80% (case 25); and (**c**) 85% (case 26).

For the flow rate ratio of 0:0:100, i.e., case 5, that showed the lowest PM_10_ concentration among cases 5–24, the PM_10_ concentration in the cabin was predicted while changing the filter efficiency from 70% to 80% or 85%. The results are shown in Figure 11. It was predicted that the PM_10_ concentration in the cabin could be maintained below 35 μg/m^3^ (Figure 8 and Figure 11c) under the following two conditions. First, an airflow rate of 250 CMH should be supplied only through supply inlet 3 of each HVAC system. Second, filters with 85% efficiency should be used. This means that a filter grade that is lower than that presented for case 4 can be applied. When cooling and heating are considered, however, passengers in the center of the cabin may feel cold or hot if air is supplied only to both ends of the cabin as in case 26. Therefore, it is practically difficult to consider case 26.

### 3.4. Effect of the Total Ventilation Flow Rate on the PM_10_ Concentration Distribution

**Figure 12:**
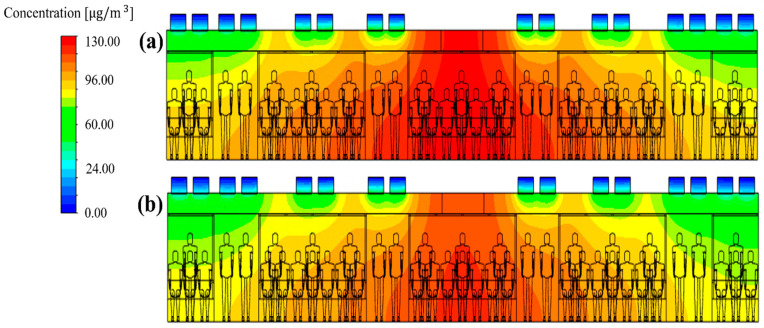
PM_10_ concentration distribution in the subway cabin according to the total ventilation flow rate (filter efficiency of 70% and flow rate ratio of 20:20:60 applied): (**a**) 600 CMH (case 27); (**b**) 700 CMH (case 28).

**Figure 13:**
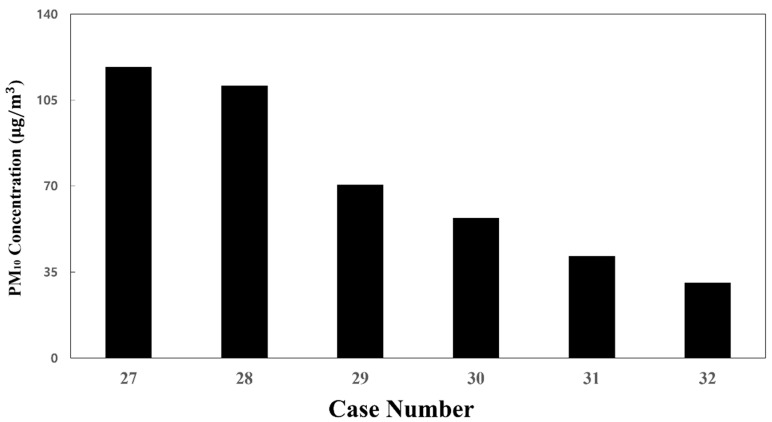
Average PM_10_ concentration in the cabin at a fixed flow rate ratio of 20:20:60 (total ventilation flow rate of 600 CMH applied for cases 27, 29, and 31, 700 CMH—for cases 28, 30, and 32; filter efficiency of 70% applied for cases 27 and 28, 80%—for cases 29 and 30, 85%—for cases 31 and 32).

**Figure 14:**
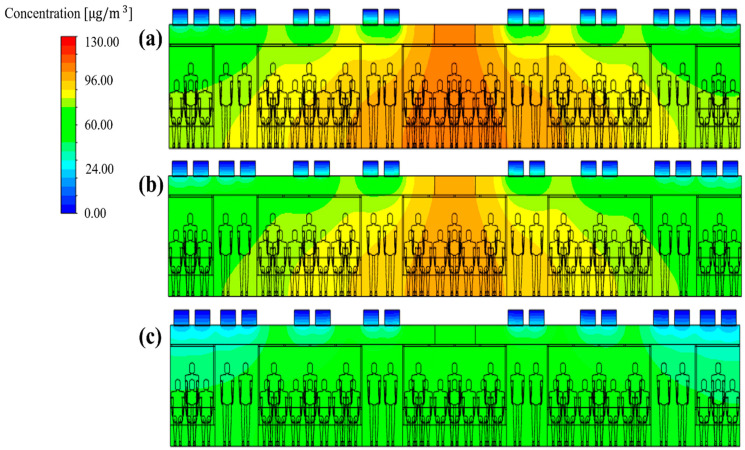
PM_10_ concentration distribution in the subway cabin according to the total ventilation flow rate (filter efficiency of 80% and flow rate ratio of 20:20:60 applied): (**a**) 500 CMH (case 2); (**b**) 600 CMH (case 29); (**c**) 700 CMH (case 30).

**Figure 15:**
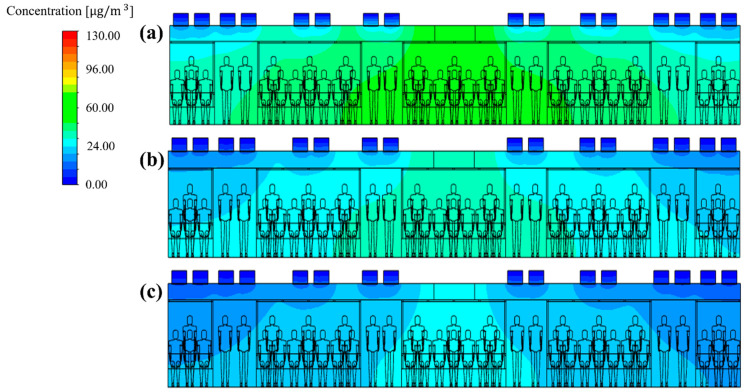
PM_10_ concentration distribution in the subway cabin according to the total ventilation flow rate (filter efficiency of 85% and flow rate ratio of 20:20:60 applied). (**a**) 500 CMH (case 3); (**b**) 600 CMH (case 31); and (**c**) 700 CMH (case 32).

Next, the total ventilation flow rate of the HVAC systems equipped with a SCAP was increased. Meanwhile, the flow rate ratio of supply inlets 1, 2, and 3 was maintained at 20:20:60, corresponding to the reference model (case 1), for more even air supply from the center to both ends of the cabin. Figure 10a and Figure 12a,b compare the PM_10_ concentration distribution in the cabin for the total ventilation flow rates of 500, 600, and 700 CMH, respectively, for the same flow rate ratio of 20:20:60 and filter efficiency of 70%. Compared to the total ventilation flow rate of 500 CMH (case 1), the PM_10_ concentration decreased by approximately 6% for 600 CMH (case 27) and 20% for 700 CMH (case 28), but the average PM_10_ concentration in the cabin could not meet the condition of ≤35 μg/m^3^, as shown in Figure 13. This means that the filter efficiency of 70% has a limit. As the next step, the filter efficiency was increased to 80%. Figure 14a–c displays the PM_10_ concentration distribution in the cabin for the total ventilation flow rates of 500, 600, and 700 CMH, respectively, for the same flow rate ratio of 20:20:60 and filter efficiency of 80%. Compared to the total ventilation flow rate of 500 CMH (case 2), the PM_10_ concentration decreased by approximately 10% for 600 CMH (case 29) and 27% for 700 CMH (case 30), but the average PM_10_ concentration in the cabin was still higher than 35 μg/m^3^ as shown in Figure 13. Therefore, the filter efficiency was further increased to 85%, and the PM_10_ concentration in the cabin was predicted according to the total ventilation flow rate. Figure 15a–c shows the PM_10_ concentration distribution in the cabin for the total ventilation flow rates of 500, 600, and 700 CMH, respectively, while maintaining the filter efficiency and flow rate ratio at 85% and 20:20:60, respectively. Compared to the ventilation flow rate of 500 CMH (case 3), the average PM_10_ concentration decreased by approximately 11% for 600 CMH (case 31) and by 36% for 700 CMH (case 32). In particular, as displayed in Figure 13, when filters with 85% efficiency were used in the HVAC systems equipped with a SCAP and the total ventilation flow rate was set to 700 CMH with the flow rate ratio of 20:20:60, it was predicted that the average PM_10_ concentration in the cabin could be maintained below 35 μg/m^3^.

## 4. Conclusions

In this study, the PM_10_ concentration distribution in a subway cabin was analyzed according to the filter efficiency applied to the HVAC systems equipped with a SCAP, ventilation flow rate, and flow rate ratio. In this way, the optimum conditions were determined to meet the PM_10_ concentration maximum inside subway cabins (≤35 μg/m^3^) as reduced by the city of Seoul. A simulation was performed by simplifying a subway cabin operated on Seoul Metro Line 5, and the simulation method used in this study was verified through a comparison with the experiment results obtained by Jeong et al. [18]. Under the current subway train HVAC operating conditions of case 1 (filter efficiency of 70%, total ventilation flow rate of 500 CMH, and flow rate ratio of 20:20:60), the PM_10_ concentration was highest in the central area of the cabin where the exhaust outlets are located. It decreased toward both ends of the cabin. When the total ventilation flow rate and the flow rate ratio were constant, it was found that the PM_10_ concentration in the cabin could be maintained below 35 μg/m^3^ by increasing the filter efficiency to 90% (case 4). The supply inlets were divided into three areas, and the PM_10_ concentration in the cabin was identified while changing the flow rate ratio between the areas. The results show that the PM_10_ concentration in the cabin decreased with an increasing flow rate through supply inlet 3 located at both ends of the cabin because the airflow throughout the cabin became efficient. In case 5 where all supply air was introduced only through supply inlet 3, the lowest PM_10_ concentration was predicted. Under the total ventilation flow rate of 500 CMH and the flow rate ratio of 0:0:100, the PM_10_ concentration in the cabin could be maintained below 35 μg/m^3^ by increasing the filter efficiency to 85% (case 26). However, a certain amount of supplied air may be required in the central area of the cabin for the thermal comfort of the passengers. Thus, it was deemed appropriate to maintain the flow rate ratio of the reference case, i.e., 20:20:60, which represents the current subway train operating conditions. Therefore, the total ventilation flow rate was increased while maintaining the filter efficiency of 85% and the flow rate ratio of 20:20:60. It was found that the PM_10_ concentration in the cabin could be maintained below 35 μg/m^3^ by increasing the total ventilation flow rate to 700 CMH (case 32). The results of this study are expected to be used as important reference data for improving indoor air quality in subway cabins. It should be noted that the results of this study have some limitations. In other words, the simulation did not consider the effect of heat generation by the passengers, and the effect of the proposed conditions on PM_10_ concentration reduction were not experimentally verified. Therefore, future studies need to be performed to experimentally measure the PM_10_ concentration in subway cabins by operating HVAC systems under the proposed conditions. In the future, it will also be necessary to analyze energy efficiency and thermal comfort considering the temperature and humidity distribution in addition to the PM concentration distribution. For the thermal comfort analysis, the buoyancy effect due to heat generation by passengers and the effect of seasonal operation of HVAC systems (i.e., heating or cooling) need to be included.

## Figures and Tables

**Table 1:** PM_10_ concentration measurements in subway cabins and tunnels of Seoul Metro Line 5 during non-rush hours (adapted from Jeong et al. [18]).

Location	PM_10_ Concentration (μg/m^3^)	
Subway cabin	Avg.	93.1
	Max.	138.4
	Min.	68.0
Subway tunnel	Avg.	161.1
	Max.	226.8
	Min.	114.1

**Table 2:** All the cases considered in this study with varying the parameters of the total ventilation flow rate, filter efficiency, and flow rate ratio.

Case Number	Total Ventilation Flow Rate (CMH)	Filter efficiency (%)	Flow Rate Ratio (%)		
			Supply Inlet 1	Supply Inlet 2	Supply Inlet 3
Case 1	500	70	20	20	60
Case 2	500	80	20	20	60
Case 3	500	85	20	20	60
Case 4	500	90	20	20	60
Case 5	500	70	0	0	100
Case 6	500	70	0	20	80
Case 7	500	70	0	40	60
Case 8	500	70	0	60	40
Case 9	500	70	0	80	20
Case 10	500	70	0	100	0
Case 11	500	70	20	0	80
Case 12	500	70	20	40	40
Case 13	500	70	20	60	20
Case 14	500	70	20	80	0
Case 15	500	70	40	0	60
Case 16	500	70	40	20	40
Case 17	500	70	40	40	20
Case 18	500	70	40	60	0
Case 19	500	70	60	0	40
Case 20	500	70	60	20	20
Case 21	500	70	60	40	0
Case 22	500	70	80	0	20
Case 23	500	70	80	20	0
Case 24	500	70	100	0	0
Case 25	500	80	0	0	100
Case 26	500	85	0	0	100
Case 27	600	70	20	20	60
Case 28	700	70	20	20	60
Case 29	600	80	20	20	60
Case 30	700	80	20	20	60
Case 31	600	85	20	20	60
Case 32	700	85	20	20	60

## Data Availability

Not applicable.

## References

[1] Katsouyanni K. (2003). Ambient air pollution and health. Br. Med. Bull..

[2] Lee E.S., Lee T.J., Park M.B., Park D.S., Kim D.S. (2017). Characteristics of particulate matter concentration and classification of contamination patterns in the Seoul metropolitan subway tunnels. J. Korean Soc. Atmos. Environ..

[3] Kim J.B., Woo S.H., Hwang M.S., Yoon H.H., Jung J.S., Bae G.N. (2017). Change in PM10 concentration by train operation in a single tunnel section. J. Korean Soc. Urban Railw..

[4] Mao P., Li J., Xiong L., Wang R., Wang X., Tan Y., Li H. (2019). Characterization of urban subway microenvironment exposure—A case of Nanjing in China. Int. J. Environ. Res. Public Health.

[5] Baudet A., Baurès E., Blanchard O., Le Cann P., Gangneux J.P., Florentin A. (2022). Indoor carbon dioxide, fine particulate matter and total volatile organic compounds in private healthcare and elderly care facilities. Toxics.

[6] Cournane S., Conway R., Byrne D., O’Riordan D., Silke B. (2016). Air quality and hospital outcomes in emergency medical admissions with respiratory disease. Toxics.

[7] Kim J.B., Kim S., Lee G.J., Bae G.N., Cho Y., Park D., Lee D.H., Kwon S.B. (2014). Status of PM in Seoul metropolitan subway cabins and effectiveness of subway cabin air purifier (SCAP). Clean Technol. Environ. Policy.

[8] Park S., Kim M., Namgung H.G., Kwon S.B. (2017). A study on HVAC performance improvement through fine dust blocking device. IJAR.

[9] Son Y.S., Oh Y.H., Choi I.Y., Dinh T.V., Chung S.G., Lee J.H., Park D., Kim J.C. (2019). Development of a magnetic hybrid filter to reduce PM10 in a subway platform. J. Hazard. Mater..

[10] Park H., Kim W., Jo Y. (2013). Field application of a double filtration process to control fine dust in a metro subway station. J. Korean Soc. Atmos. Environ..

[11] Kwon S.B., Song J.H., Ryu J.H., Jo S.W., Oh T.S., Bae S.J., Kim H.G. (2015). A study on the improvement of the air exhaust system at the PSD installed subway station. J. Korean Tunn. Undergr. Space Assoc..

[12] Kwon S.B., Park D.S., Cho Y., Kim J.B., Kim T. (2012). Mechanical ventilation strategy for subway cabins using numerical simulations. J. Civ. Eng. Archit..

[13] Zhao L., Zhou H., Jin Y., Li Z. (2022). Experimental and numerical investigation of TVOC concentrations and ventilation dilution in enclosed train cabin. Building Simulation.

[14] Kwon S.B., Park D.S., Cho Y.M., Kim J.B., Cho G.H., Nam G.S., Lee J.Y., Kim T.S. Removal efficiency of PM_10_ & CO_2_ in subway mock-up cabin. Proceedings of the Korean Society for Railway Conference.

[15] Kim J.B., Kwon S.B., Park D.S., Cho Y., Namgoong S., Han T.W., Cho K., Kim T. Efficiency of dust removal device in subway cabin. Proceedings of the 7th Asian Aerosol Conference.

[16] Chang Z., Yi K., Liu W. (2021). A new ventilation mode of air conditioning in subway vehicles and its air distribution performance. Energy Built Environ..

[17] Nazari A., Hong J., Taghizadeh-Hesary F., Taghizadeh-Hesary F. (2021). Reducing Virus Transmission from Heating, Ventilation, and Air Conditioning Systems of Urban Subways. https://www.researchsquare.com/article/rs-1164057/v1.

[18] Jeong W., Lee Y., Choi K., Park D. (2016). Particulate matters levels in subway tunnels and cabins. Int. J. Environ. Monit. Anal..

[19] Chang T.B., Sheu J.J., Huang J.W., Lin Y.S., Chang C.C. (2018). Development of a CFD model for simulating vehicle cabin indoor air quality. Transp. Res. D: Transp. Environ..

[20] Bolourchi A., Atabi F., Moattar F., Ehyaei M.A. (2018). Experimental and numerical analyses of particulate matter concentrations in underground subway station. Int. J. Environ. Sci. Technol..

[21] ANSYS FLUENT Theory Guide, Release 15.0, November 2013. http://www.pmt.usp.br/academic/martoran/notasmodelosgrad/ANSYS%20Fluent%20Theory%20Guide%2015.pdf.

[22] Cao Q., Liu M., Li X., Lin C.H., Wei D., Ji S., Zhang T., Chen Q. (2022). Influencing factors in the simulation of airflow and particle transportation in aircraft cabins by CFD. Build. Environ..

[23] Li M., Yan Y., Zhao B., Tu J., Liu J., Li F., Wang C. (2018). Assessment of turbulence models and air supply opening models for CFD modelling of airflow and gaseous contaminant distributions in aircraft cabins. Indoor Built Environ..

[24] Lin Z., Chow T.T., Tsang C.F., Fong K.F., Chan L.S. (2005). CFD study on effect of the air supply location on the performance of the displacement ventilation system. Build. Environ..

[25] Posner J.D., Buchanan C.R., Dunn-Rankin D. (2003). Measurement and prediction of indoor air flow in a model room. Energy Build..

